# Vector-based analysis of cortical activity associated with dumbbell exercise using functional near-infrared spectroscopy

**DOI:** 10.3389/fspor.2022.838189

**Published:** 2022-09-12

**Authors:** Toshinori Kato

**Affiliations:** Department of Brain Environmental Research, KatoBrain Co., Ltd., Tokyo, Japan

**Keywords:** phase, motor, brain, function, vector analysis, near-infrared spectroscopy, fNIRS, dumbbell exercise

## Abstract

The mechanisms *via* which the brain and muscles work together remain poorly understood. The use of vector-based fNIRS, to propose a new metric and imaging method to understand neural activation during dumbbell-lifting exercises. This method can simultaneously measure oxyhemoglobin (oxyHb) and deoxyHb levels so that the angle *k*: Arctan (deoxyHb/oxyHb) represents the degree of oxygen exchange in the brain and can be used to quantify the distribution of oxygen consumption. The amplitude L of the vector reflects the intensity of the response caused by the amount of change in Hb. This study used vector-based fNIRS to simultaneously measure the left primary motor cortex (left M1), multiple peripheral regions, and the right biceps brachii muscle. The subjects were seven healthy adults. The task was a dumbbell-lifting exercise involving flexion and extension of the elbow joints of both arms. Dumbbell weights of 0 (no dumbbell), 4.5, and 9.5 kg were used. During dumbbell exercise, oxygen exchange increased in the left M1, indicating increased local oxygen consumption. Around the left M1, the cerebral oxygen exchange decreased, and oxygen supply increased without cerebral oxygen consumption. The spatial agreement between the maximum value of oxygen exchange *k* and L during the task was <20%. Therefore, the dumbbell-lifting exercise task study reported here supported the hypothesis that cerebral oxygen consumption associated with neural activation does not coincide with the distribution of cerebral oxygen supply. The relationship between the brain oxygen supply from the site of increased oxygen exchange in the brain and its surrounding areas can be quantified using the vector method fNIRS.

## Introduction

Studies of brain oxygenation during high-intensity exercise (Rupp and Perrey, 2008) and walking (Khan et al., 2021) have been performed using functional near-infrared spectroscopy (fNIRS). Physical activity induces local cerebral oxygen consumption and demand for oxygen supply. However, conventional brain functional imaging currently does not include a method for distinguishing oxygen consumption from oxygen supply simultaneously. Thus, the spatial distribution between oxygen consumption and supply in the brain that accompanies exercise remains largely unknown.

In fNIRS, variations in the ratio between changes in oxyhemoglobin (oxyHb) and deoxyHb are observed according to the site, thus reflecting the spatial distribution of oxygen consumption and supply. It is known that fNIRS is capable of measuring the fast oxygen response in capillary events (FORCE) associated with neuronal responses, defined as the FORCE effect (Kato, 2004).

The FORCE effect indicates tissue hypoxia with an increase in deoxyHb and a decrease in oxyHb, reflecting increased oxygen consumption. It has been reported that a temporary FORCE occurred in the primary motor cortex (M1) at the onset of a hand grasping task, and deoxyHb decreased and oxyHb increased around M1, reflecting oxygen supply (Akiyama et al., 2006). In a task that activated the supplementary motor area (SMA), deoxyHb and oxyHb were increased in the SMA. OxyHb increased and deoxyHb fell slightly in the sensory-motor cortex and the pre-SMA (Hatakenaka et al., 2007). Several reports have shown that oxyHb analysis alone cannot accurately detect the localization of neuroactivation (Cyranoski, 2011; Takahashi et al., 2011) and that functional images of increasing oxyHb may differ from the actual distribution of oxygen consumption (Kato, 2018, 2021).

Detecting changes in early deoxygenation has been considered a more useful spatial indicator of neuronal activity than increases in oxyHb or cerebral blood volume (CBV) because they occur in a more limited area (Ances, 2003; Kato, 2004; Khan et al., 2021). However, the oxygen consumption and supply triggered by neuroactivation cannot be evaluated simultaneously based on deoxyHb alone. In terms of technological advances, earlier NIRS was used as a cerebral oxygen monitor that could be measured over the scalp, and it was not until July 1991 that it was first successfully demonstrated that fNIRS could detect regional brain function (Kato, 2018). In addition, there was a lack of theory to quantitatively integrate neural activation from multiple sites (channels) and indices (Kato et al., 1993; Ferrari and Quaresima, 2012).

A vector-based model of cerebral oxygen regulation (CORE model; Kato, 2006, 2013) can explain variations in the ratios of concentration changes in oxyHb and deoxyHb without exception. Vector-based fNIRS allows the integration of the dynamics of oxyHb and deoxyHb changes in multiple channels on the same vector coordinates, thus enabling the detection of oxygen dynamics as differences between CORE vectors. Oxygen consumption associated with neuroactivation can be classified into one of eight phases according to the combination of the CORE vector components, i.e., oxyHb, deoxyHb, CBV, and cerebral oxygen exchange (COE). Using vector-based fNIRS, the patterns of FORCE in the language area were analyzed and classified into five levels based on variations in oxygen consumption (Yoshino and Kato, 2012).

A technique has been proposed for detecting and imaging the spatial distribution of neuroactivation by differentiating the phase distributions of oxygen consumption and oxygen supply (Kato et al., 2003; Kato, 2018). Temporal FORCE effects were reported to be detected 2–3 s after the onset of the stimulus task. Therefore, it was hypothesized that when neural activity is strongly induced, the FORCE effect occurs over a larger spatial area and for a longer period of time. Furthermore, it is assumed that the oxygen consumption and oxygen supply distributions do not coincide with the oxygen consumption distribution due to neural activation during exercise when the FORCE effect strongly appears. As a result, when oxygen consumption increases in the M1 and a strong FORCE effect is induced, a mechanism to supply fresh blood to the area around M1 could be detected. Therefore, the task of lifting heavier dumbbells has been chosen, which could be predicted to elicit strong neural activity in M1.

To identify differences in the strength of neuroactivation, measurements were taken from the M1 and surrounding sites using vector-based fNIRS during exercise tasks using three different dumbbell weights and report a new imaging method for differentiating between oxygen consumption and supply.

## Materials and methods

### Subjects

The subjects of this study were seven healthy adults: six males and one female; the mean age was 33.3 ± 8.0 years. The participants had some experience in sports before age 20, but had not received any intensive strength training for at least 1 year.

All participants were identified as being right-handed based on the Edinburgh Handedness Inventory. The study was explained to the subjects in writing and orally, and they provided prior written consent for participation in and reporting of this study.

The experimental procedure complied with the principles of the Declaration of Helsinki. All subjects received full explanation of the procedures and provided written informed consent for participation in the study. This study was reviewed and approved by the Ethics Committee of KatoBrain Co., Ltd. which included outside members.

### fNIRS measurement

A multi-channel NIRS apparatus (ETG-100) monitored localized changes in oxyHb and deoxyHb. The scalp was irradiated at each site with two wavelengths of near-infrared light (780 and 830 nm) from a semi-conductor laser using three optical fibers. Three avalanche photodiodes detected light passing through the head. Hb concentration measurements were recorded at a sampling rate of 10 Hz.

**Figure 1:**
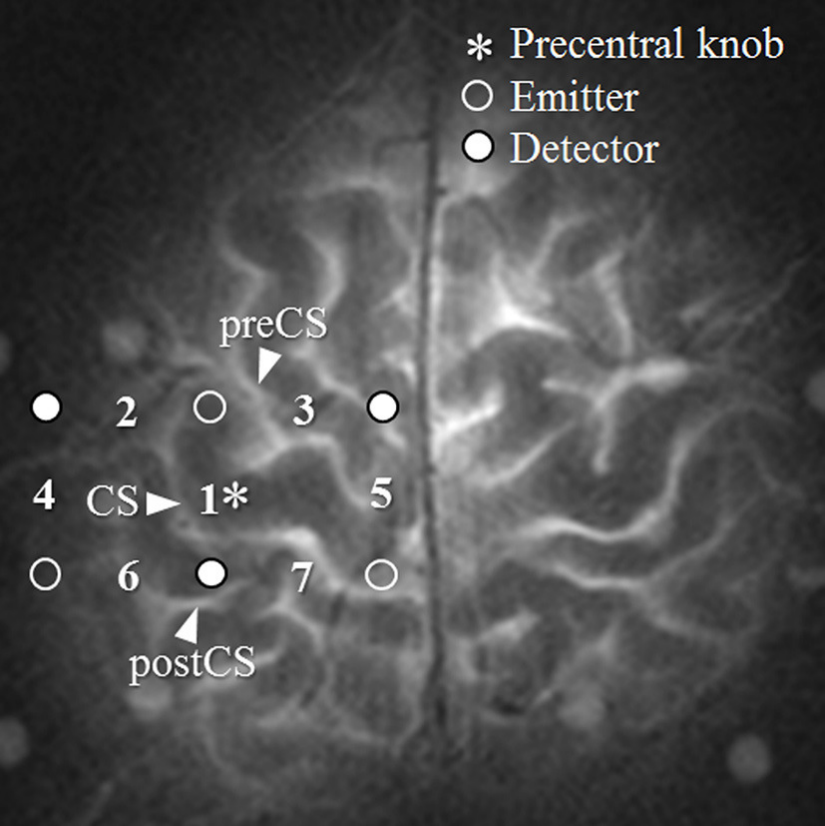
Region of measurement. The numbers between the detectors and emitters are channel numbers. CS, central sulcus.

Measurements were collected from a total of seven channels (Figure 1). Channel 1 was positioned directly over the left M1, and the remaining six channels were arranged around it: channel 2 lateral to the precentral knob, channel 3 in the premotor area, channel 5 in the SMA, and channels 4, 6, and 7 over the postcentral gyrus. The distance between emitter and detector probes was 30 mm. The M1 was identified using a technique for pre-measurement identification of target cortex sites that utilizes the distance between points of reference on the scalp from the subject's MRI (Murakoshi and Kato, 2006). The right M1 was measured by fNIRS simultaneously with the left M1. However, the analysis was limited to the left-hemisphere channel.

### Experimental procedure

The task used in this study to investigate the effects of the strength of neural activity was a dumbbell-lifting exercise that involved flexing and extending the elbow joints of both arms. It has been reported that movement-related cortical potentials increase more for isometric contractions of the elbow flexors under a heavy load than they do under a light load (Oda et al., 1996). Therefore, there were three different weights: 0 kg (no dumbbell), and dumbbells weighing 4.5 and 9.5 kg. One trial consisted of flexing and extending a given weight 12 times. Repetitions were paced at 3 s (Tamaki et al., 1994), for a total of 36 s per trial, with a 60 s resting period between trials. Each repetition lasted for 1.5 s, for a total of 3 s. Five trials with each weight made up a trial set. After completing a trial set for one weight, the subject went on to the next, heavier weight. The training order was always from lightest to heaviest, with participants always set to be most fatigued on the heaviest trial set.

For the 0 and 4.5 kg tasks, a total of 35 trials performed by the seven subjects were used for analysis. For the 9.5 kg task, one subject completed only one trial and another subject only four because the weight was too heavy, and a total of 30 trials were used for analysis.

The weight of the dumbbells that can be lifted could be different for each individual.

However, the neural activity to M1 and its surrounding sites is expected to be higher with the dumbbell weighing more. Therefore, instead of selecting the dumbbell weight that produces maximal voluntary muscle contraction, a uniform dumbbell weight was set.

### Theory of vector-based analysis

#### A vector-based model of CORE

The CORE model (Kato, 2004, 2006, 2013) can simultaneously detect seven indices reflecting the oxygen consumption and supply caused by neuroactivation. OxyHb and deoxyHb have different chemical properties (paramagnetic or diamagnetic) arising from differences in the bonding of oxygen molecules (Pauling and Coryell, 1936). The axes oxyHb (Δ*O*) and deoxyHb (Δ*D*) define an orthogonal vector coordinate plane. Rotating this Δ*O*/Δ*D* vector plane by 45° counterclockwise results in an orthogonal vector coordinate plane comprising a (Δ*O* + Δ*D*) axis and a (Δ*D* –Δ*O*) axis. The (Δ*O* + Δ*D*) vector can be defined as the CBV vector (Δ*CBV*), and the (Δ*D* –Δ*O*) vector can be considered as the COE vector (Δ*COE*).

The relationship among the four axes, Δ*O*, Δ*D*, Δ*CBV*, and Δ*COE*, is described by the following square matrix:


(1) $$\begin{array}{l} \left(\begin{array}{c} \Delta O+\Delta D\\ -\Delta O+\Delta D \end{array}\right)=\left(\begin{array}{cc} 1 & 1\\ -1 & 1 \end{array}\right)\left(\begin{array}{c} \Delta O\\ \Delta D \end{array}\right)=\left(\begin{array}{c} \Delta CBV\\ \Delta COE \end{array}\right) \end{array}$$

(2) $$\begin{array}{l} \left(\begin{array}{c} \Delta O\\ \Delta D \end{array}\right)=\frac{1}{2}\left(\begin{array}{cc} 1 & -1\\ 1 & 1 \end{array}\right)\left(\begin{array}{c} \Delta CBV\\ \Delta COE \end{array}\right) \end{array}$$

**Figure 2:**
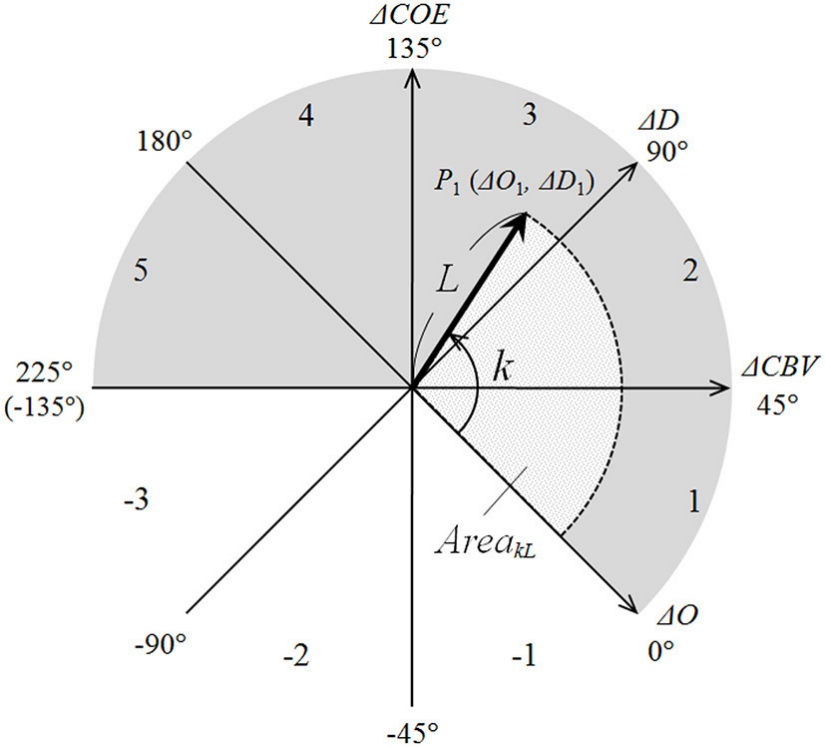
CORE vector coordinates. Values for *k* (in degrees) are shown at the tips of the axes. Phase numbers are shown in each octant arc.

The polar coordinate plane comprising these four axes is termed as the CORE vector plane and the vector tracks on the polar coordinate as CORE vectors (Figure 2). The vectors have four components, i.e., Δ*O*, Δ*D*, Δ*CBV*, and Δ*COE*, and the analysis of the CORE vectors allows handling these four Hb indices as the components of a wave.

On the CORE vector plane, a positive value for Δ*CBV* indicates increasing Δ*CBV* and a negative value for Δ*CBV* indicates decreasing Δ*CBV*; moreover, a positive value for Δ*COE* indicates increasing Δ*COE* and shows hypoxic change, and a negative value for Δ*COE* indicates decreasing Δ*COE* and shows hyperoxic change. In other words, Δ*COE* reflects increasing or decreasing cerebral oxygen extraction from the capillaries to the cells.

#### Phase components of CORE vectors

The phases on the CORE vector plane are quantitatively defined indices of the degree of oxygen exchange that reflect the strength of oxygen consumption. Phase is defined by the angle *k*, the ratio of Δ*D* to Δ*O*. *k* is the angle between a CORE vector and the positive Δ*O* axis and is determined as follows:


(3) $$\begin{array}{l} k=\;Arc\;\tan\left(\frac{\Delta D}{\Delta O}\right)=Arc\;\tan\left(\frac{\Delta COE}{\Delta CBV}\right)+\;45^{{}^{\circ}}\;\\ \left(-135^{{}^{\circ}}\;≦\;k\;≦\;225^{{}^{\circ}}\right) \end{array}$$

It is a quantitative index indicating the degree of oxygen consumption over a certain period. *k* = 0° is on the positive Δ*O* axis and coincides with the oxygen density of arterial blood. Thus, an increase or decrease in *k* indicates a change in oxygen density. The eight octants of 45° on the vector plane in Figure 1 can be classified into activation phases (Phases 1 through 5) and non-activation phases (Phases −1 through −3). Phase 1 (0 < Δ*D* < Δ*O*; Δ*COE* < 0 < Δ*CBV*) and Phase 2 (0 < Δ*O* < Δ*D*; 0 < Δ*COE* < Δ*CBV*) are canonical activation, showing increases in both Δ*D* and Δ*O*. Phase 3 (Δ*O* < 0 < Δ*D*; 0 < Δ*CBV* < Δ*COE*) is hypoxic–hyperemic activation, showing a decrease in Δ*O* together with an increase in ΔCBV. Phase 4 (Δ*O* < 0 < Δ*D*; Δ*CBV* < 0 < Δ*COE*) and Phase 5 (Δ*O* < Δ*D* < 0; Δ*CBV* < 0 < Δ*COE*) are hypoxic–ischemic activation, showing an increase in Δ*COE* together with a decrease in Δ*CBV*. Conversely, Phases −1 through −3 indicate non-activation and show decreases in both Δ*D* and Δ*COE*. Oxygen consumption during neuroactivation can be considered higher during the activation phases vs. the non-activation phases.

#### The scalar *L*

The scalar *L* between point P_1_ (Δ*O*_1_, Δ*D*_1_) and the origin can be described by the following equation:


(4) $$L\;=\;\sqrt{{\left(\Delta O_{1}\right)}^{2}+{\left(\Delta D_{1}\right)}^{2}}\;=\;\frac{1}{\sqrt{2}}\sqrt{{\left(\Delta COE_{1}\right)}^{2}+{\left(\Delta CBV_{1}\right)}^{2}},$$

where *L* represents the intensity of Hb changes (oxyHb and deoxyHb); i.e., the scalar component *L* of the CORE vector also includes Δ*CBV* and Δ*COE*.

The contribution percentage of Δ*O* and Δ*D* to *L* is calculated by squaring both sides of Equation (4).


(5) $$\begin{array}{l} 1={\left(\frac{\Delta O}{L}\right)}^{2}\;+{\left(\frac{\Delta D}{L}\right)}^{2} \end{array}$$

(6) $$\begin{array}{l} \text{Percentage contributed by }\Delta \text{O}:{\left(\frac{\Delta O}{L}\right)}^{2}\;\times \;100\;\left(\%\right) \end{array}$$

(7) $$\begin{array}{l} \text{Percentage contributed by }\Delta \text{D}:{\left(\frac{\Delta D}{L}\right)}^{2}\;\times \;100\;\left(\%\right) \end{array}$$

The following relationship exists between *L* and the angle *k*:


(8) $$\begin{array}{l} \left(\begin{array}{c} \Delta COE\\ \Delta CBV \end{array}\right)=L\left(\begin{array}{c} \cos k\\ \sin k \end{array}\right) \end{array}$$

In this way, CORE vectors have properties of waves: phase (*k*) and intensity (*L*). The imaging of *k* indicates the phase distribution of oxygen consumption, whereas the imaging of Δ*O* shows the distribution of oxygen supply. The distribution of the percentages of contribution by Δ*O* indicates the relative contribution of Δ*O* that is affected by changes in *k* with respect to *L*.

#### Oxygen regulation index

A new index was created to reflect phase and amplitude (*L*). The area created by the rotational motion of the CORE vector is defined on the vector plane using *L*_t_ and the radian of *k* ($k_{\text{t}}^{\text{rad}}$), as follows:


(9) $$\begin{array}{l} {\;Area}_{kL}=\;\overset{\text{n}}{\underset{\text{n}=1}{\sum }}\frac{1}{2}\;{k^{\text{rad}}}_{t}\;•\;{L_{\text{t}}}^{2}, \end{array}$$

where n is the number of trials analyzed for all subjects An *Area*_*kL*_ is a cumulative area encompassed by the CORE vector track; that is, the area swept out by *L*_t_ over the range of *k*_t_ degrees from the positive Δ*O* axis (*k* = 0°) during *t* seconds from task initiation. A positive value for *Area*_*kL*_ indicates an area increasing in the direction of increased oxygen exchange degree (0° < *k*), and a negative value indicates an area increasing in the direction of increased oxygen not used for oxygen exchange (*k* < 0°).

### Vector-based analysis

Measurements performed over 36 s during a trial and 36 s after a trial were used for analysis. Functional imaging was performed using seven indices: the four-vector components (Δ*O*, Δ*D*, Δ*CBV*, and Δ*COE*) of the addition vectors during the tasks, phase (*k*), the scalar (*L*), and the oxygen regulation index (*Area*_*kL*_).

The time-course changes were averaged, with each trial onset set as zero. The time-course changes in ΔCBV and ΔCOE were calculated from the time-course changes in ΔO and ΔD using Equations (1) and (2). Vector tracks were plotted using task onset as the origin and the grand averages calculated for all trials for each component. No baseline normalization or motion correction was performed as a preprocessing step for the analysis, as these may distort the phase of oxygen exchange (Kato, 2021).

CORE addition vectors during and after the tasks were calculated to observe total changes across the task time. The cumulative sums of Δ*CBV* and Δ*COE* grand averages were determined, and the CORE addition vectors were plotted using those values. Imaging of each vector component (Δ*O*, Δ*D*, Δ*CBV*, and Δ*COE*) was carried out using the cumulative sums recorded during the task. Imaging of *k* and *L* was performed using Equations (3) and (4), with values determined from the CORE addition vectors. Imaging of *Area*_*kL*_ was carried out based on Equation (9) based on the cumulative sums of the area determined from *k* and *L* of the single-trial CORE addition vectors.

The phase distribution of a CORE vector was determined by first classifying *k* for each trial into the appropriate octant and then calculating the ratio of the number of trials in each phase to the total number of trials for analysis for each task. Phase distribution for the sites surrounding the left M1 was calculated by averaging the phase distributions of all surrounding channels.

Spatial concordance between *k* and *L* was calculated as the ratio of the number of trials in which the maximum values for *k* and *L* coincided with the number of trials in the analysis of each task.

The percent contribution of Δ*O* and Δ*D* to *L* was calculated using Equations (6) and (7). Values for *L* of the CORE addition vectors and the cumulative sums of Δ*D* and Δ*O* were used for the calculation. The contribution rates for the sites surrounding the left M1 were averaged from the contribution rates of all channels. Differences in contribution rates were calculated by subtracting the percentages for Δ*O* from those for Δ*D*.

### Statistics

Multiple analysis of variance and *post-hoc* multiple comparison tests (Scheffe's) were performed using Δ*CBV* and Δ*COE* for all trials to determine the significance of differences between the left M1 and the averages of the surrounding sites. Statistical tests were performed for *k, L*, and the *Area*_*kL*_, to determine the significance of differences between the left M1 and the averages of surrounding sites. Independent *t*-tests were used for *L* and the *Area*_*kL*_, and Watson's U2 test was used for *k*. Differences in phase distribution between the left M1 and the surrounding sites were compared using Fisher's exact test for each phase.

## Results

### Time courses and functional images of CORE vector components

**Figure 3:**
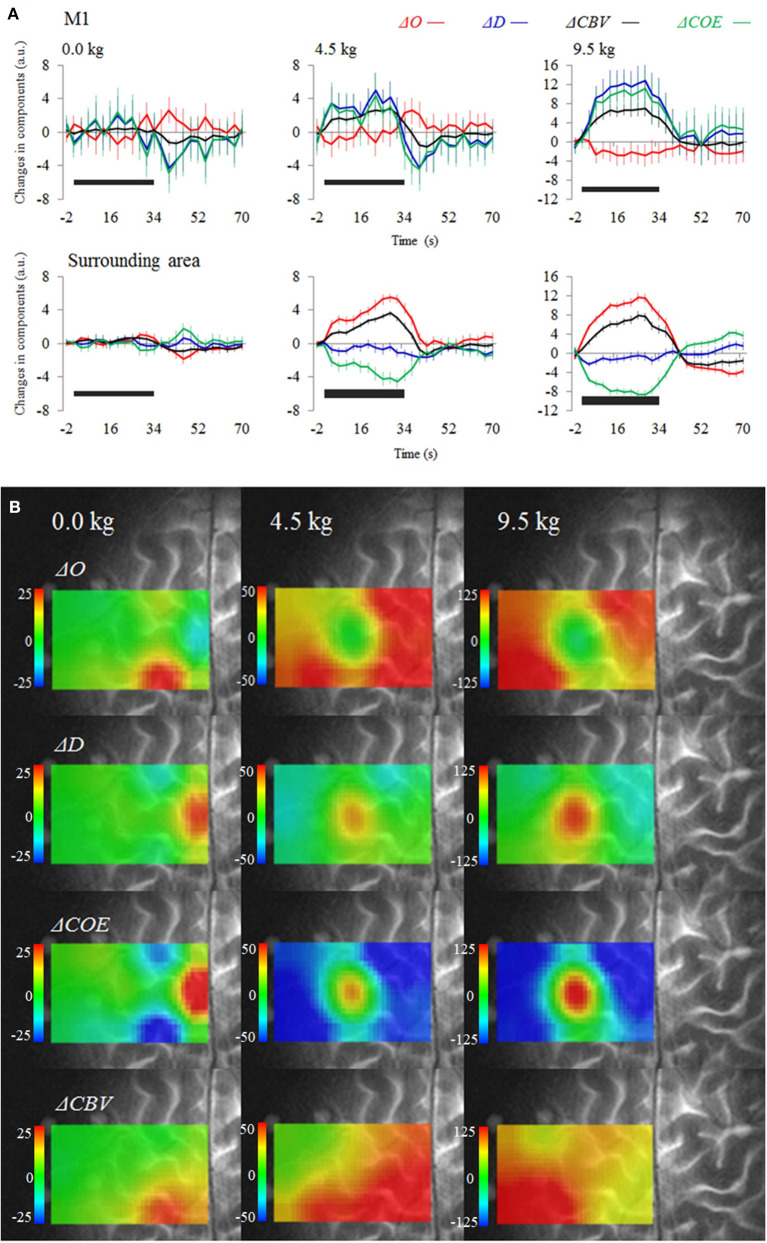
**(A)** Average time courses and standard error for the four-vector components at the left M1 and surrounding sites (averaged). **(B)** Functional images show changes in the vector components during a task. The black bars on the figure represent the period of the dumbbell exercise.

Figure 3A shows the average time courses for each vector component during and after the tasks. Figure 3B is a functional image of the four-vector components during a task. At the left M1 (channel 1), Δ*O* decreased for the 4.5 and 9.5 kg tasks, whereas Δ*D* and Δ*CBV* increased. However, in the surrounding sites, Δ*O* increased, and Δ*D* and Δ*COE* decreased. The left M1, where the maximum decrease in Δ*O* occurred, was surrounded by a high oxygen supply (increased Δ*O*), in a donut-like shape. The sites of maximum increase in Δ*D* and Δ*COE* were the left M1 for the 4.5 and 9.5 kg tasks and the SMA (channel 5) for the 0 kg task. Δ*CBV* increased in both the left M1 and the surrounding sites, not indicating localization. At 9.5 kg, the rate of the maximum increase of each vector component at the left M1 was 0% for Δ*O*, 0% for Δ*CBV*, 56.7% for Δ*D*, and 63.3% for Δ*COE*.

### Functional images using *k, L*, and the new index *Area_***kL***_*

**Figure 4:**
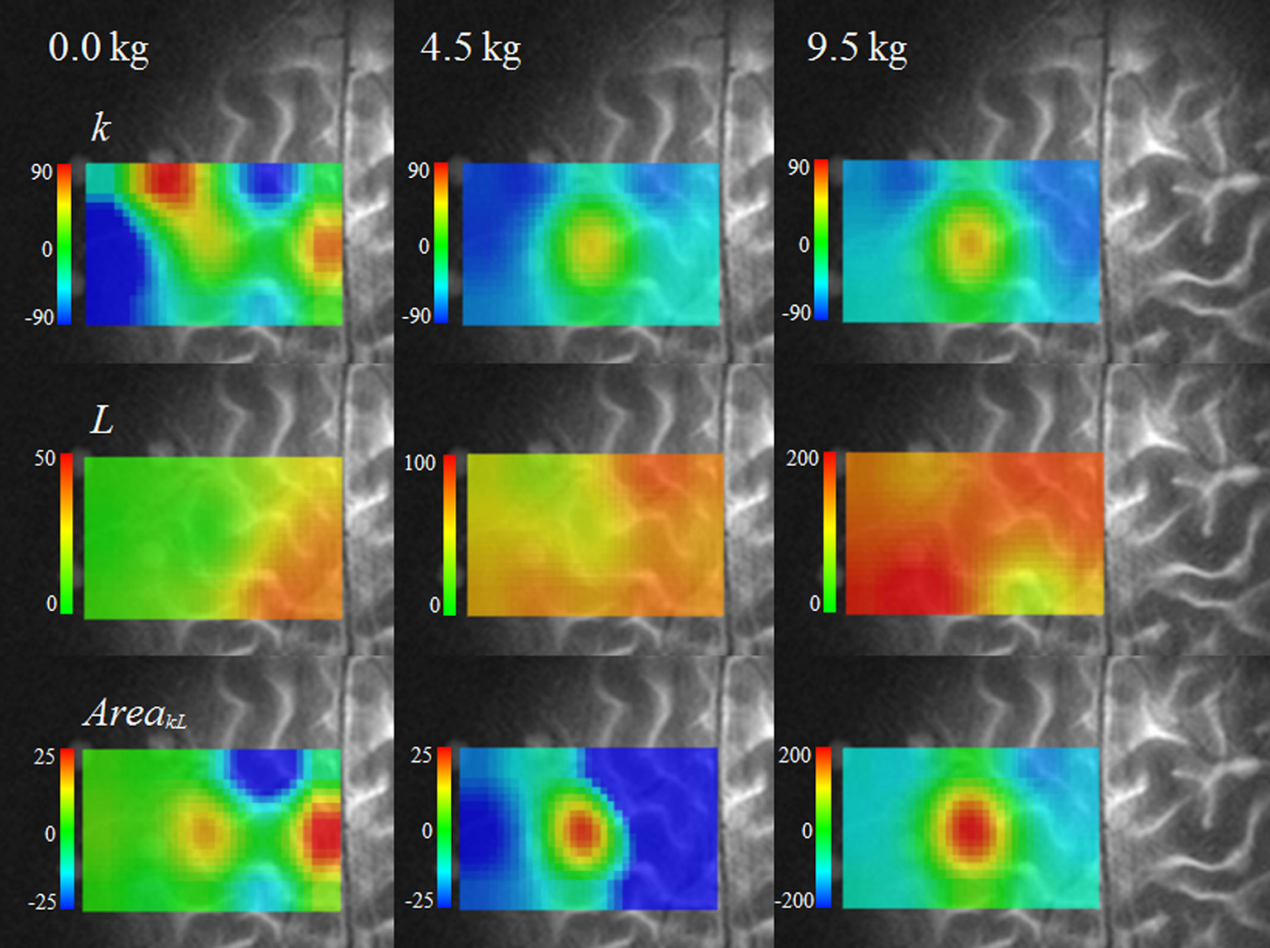
Functional images of three indices (*k, L*, and *Area*_*kL*_) during tasks.

Figure 4 provides functional images of *k, L*, and the *Area*_*kL*_. The SMA was the site with the maximum increase in k at 0 kg. At 4.5 and 9.5 kg, the left M1 showed the maximum increase in *k*, whereas oxygen consumption decreased in the surrounding area. The maximum increase in *L* appeared not at the left M1, but in the surrounding area. The spatial concordance of the maximum increases in *k* and *L* was low; 11.4% at 0 kg, 17.1% at 4.5 kg, and 20.0% at 9.5 kg.

The spatial concordance of the maximum increases in *k* and *Area*_*kL*_ increased as the dumbbell weight increased; 37.1% at 0 kg, 54.3% at 4.5 kg, and 76.7% at 9.5 kg. *Area*_*kL*_ showed a significantly greater increase at the left M1 compared with the surrounding area (4.5 and 9.5 kg) (*P* < 0.01), indicating increased oxygen consumption. The site of maximum increase in *Area*_*kL*_ was the SMA at 0 kg. The imaging of *Area*_*kL*_ emphasized the changes at the left M1 and SMA and allowed the detection of a small increase in oxygen consumption at the left M1 that could not be detected in images of the single-vector components or *L*.

### Differentiation of phase during and after motor exercise

**Figure 5:**
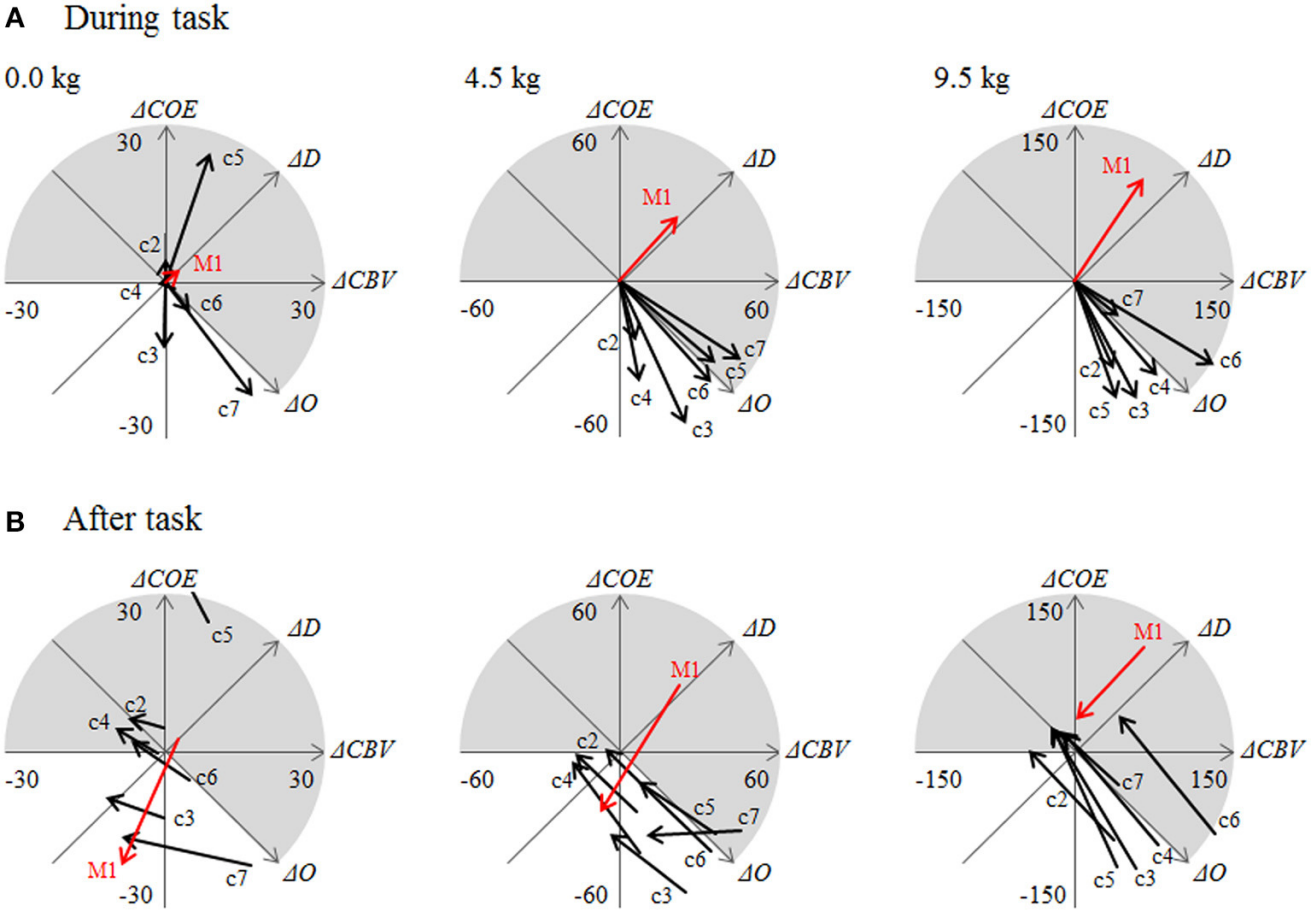
**(A)** Addition vectors during a task (0–36 s) and **(B)** after the task (36–72 s). c2 through c7 are the numbers of channels in the area surrounding the left M1, as shown in Figure 2.

Figure 5 displays the CORE addition vectors during the task (36 s) and after the task (36 s) at each channel.

Table 1 shows the phase distribution of the CORE addition vectors at the left M1 and surrounding sites.

**Table 1:** Phase distribution (%) of the CORE vectors during the tasks.

**Phase**	**0.0 kg**		**4.5 kg**		**9.5 kg**	
	**Ml**	**Surrounding area**	**Ml**	**Surrounding area**	**Ml**	**Surrounding area**
1	2.9	9.0	5.7	19.0	10.0	22.2
2	2.9	10.5	2.9	6.7	10.0	7.2
3	31.4*	16.2	54.3**	11.0	46.7**	3.9
4	5.7	12.4	5.7	4.3	0.0	0.6
5	2.9	9.5	0.0	7.1	0.0	0.0
−1	11.4	18.6	20.0	38.1*	16.7	43.3**
−2	42.9**	16.7	8.6	9.0	16.7	15.6
−3	0.0	7.1	2.9	4.8	0.0	7.2
Total (%)	100.0	100.0	100.0	100.0	100.0	100.0

The asterisks indicate significant differences between the left M1 and the surrounding sites for the same phase (*P < 0.1, **P < 0.01).

Vectors for the 4.5 and 9.5 kg tasks showed significant differences between the left M1 and the surrounding sites (*P* < 0.01). The most common phase at the left M1 was Phase 3, indicating hypoxic–hyperemic activation. The percent occurrence of Phase 3 at the left M1 was 31.4% at 0 kg, 54.3% at 4.5 kg, and 46.7% at 9.5 kg, which were all significantly higher than those detected in the surrounding sites (Table 1). At this time, *k* was 83.9° at 0 kg, 93.8° at 4.5 kg, and 101.6° at 9.5 kg, which were also significantly higher than those detected in the surrounding sites (*P* < 0.05). Vectors in the surrounding sites were significantly concentrated in Phases −1 and −2, which are non-activation phases. In the surrounding area, *k* ranged from −118.3° to 135.2° at 0 kg, from −34.0° to 11.6° at 4.5 kg, and from −25.5° to 14.0° at 9.5 kg.

After the tasks, the phase differences between the left M1 and the surrounding sites disappeared. The direction of the vectors changed and moved toward the origin.

During the task, *L* increased significantly with increased dumbbell weight at both the left M1 and the surrounding area (*P* < 0.01). In the 4.5 and 9.5 kg tasks, there was no significant difference between *L* at the left M1 and the surrounding sites. In this way, the CORE vectors showed specific phase distributions according to site and task.

### Contribution rate of Δ*O* and Δ*D* related to *L* change

Table 2 reports the relative contributions of Δ*O* and Δ*D* to *L* during the tasks. The contribution percentages from Δ*O* and Δ*D* were different for each measurement channel. At the left M1, the contribution of Δ*O* was small, at < 4.0%, whereas the contribution of Δ*D* was more than 96%, which represented a difference of ~92%.

**Table 2:** Average contribution to *L* and differences in the relative contributions of Δ*D* and Δ*O* during a task.

**Site**	**Task (kg)**	**Δ*O* (%)**	**Δ*D* (%)**	**Differences (%)**
M1	0	1.1	98.9	97.7
	4.5	0.4	99.6	99.1
	9.5	4.0	96.0	91.9
Surrounding area	0	56.4	43.6	−12.8
	4.5	87.9	12.1	−75.7
	9.5	91.9	8.1	83.8

Differences were determined by subtracting the contribution of Δ*O* from that of Δ*D*.

At the sites surrounding the left M1, the contribution rate was higher for Δ*O* than for Δ*D*. Differences in the contribution rates of Δ*O* and Δ*D* increased as the dumbbell weights became heavier: 12.8% at 0 kg, 65.8% at 4.5 kg, and 83.8% at 9.5 kg. Namely, the donut-shaped image surrounding the site of maximum increase in oxygen consumption became clearer as the dumbbell weight increased from 0 to 4.5 kg and then to 9.5 kg, reflecting the increase in oxygen supply (Δ*O* increase) in the area surrounding the M1 unaccompanied by oxygen consumption as the dumbbell weight increased.

Although at 0 kg *L* was smaller at the left M1 than it was at the surrounding sites (Figure 5), the contribution of Δ*O* at the left M1 was lower (1.1%) than it was at the surrounding sites (56.4%).

## Discussion

### Spatial distribution of COE

Differences in the distribution of oxygen consumption are quantifiable using the images of *k*, which represents the degree of oxygen exchange. During dumbbell exercises, oxygen exchange increased at the left M1, indicating a localized increase in oxygen consumption. In the area surrounding the left M1, oxygen exchange decreased, and oxygen supply increased unaccompanied by oxygen consumption. The spatial concordance between the maximum values for the degree of oxygen exchange during a task and *L*, which reflects the intensity of Hb changes, was <20%. The contribution rates of Δ*O* and Δ*D* differed according to the measurement site. Therefore, the hypothesis that the distribution of oxygen consumption associated with neural activation does not coincide with the distribution of oxygen supply was supported in the motor task study.

Interestingly, an increasing difference in the relative contribution of Δ*O* and Δ*D* in the area surrounding the M1 was found as the dumbbell weights increased, which suggests that oxygen supply in the surrounding area is regulated concerning the site of increasing oxygen consumption. Even at sites of increased oxygen consumption, the contribution of ΔO was found to be low. It is believed that neural activity and local cerebral blood flow (CBF) increase or decrease relatively correlatedly. Similarly, local CBF and local CBV are also thought to increase or decrease in correlation. However, it is thought that, in brain regions where COE has increased markedly because of a rapid and sustained increase in oxygen consumption relative to oxygen supply, there is a mechanism of cerebral blood circulation that promotes replenishment from surrounding sites with sufficient oxygen supply.

Neural activity may not always correlate with local CBV or CBF when it has a strong impact on the COE homeostasis for the whole brain, as in the results of this study. The detection of an excessive increase in Δ*CBV* rather than an increase in Δ*COE* around the near regions of rapidly increasing oxygen consumption was not previously known and therefore has not been incorporated into conventional models of cerebral circulation regulation (Roy and Sherrington, 1890; Lassen, 1959). A new model of whole brain circulation regulation is needed that can explain the mechanisms that maintain COE homeostasis for the whole brain.

Using the vector method fNIRS, it is necessary to study further how the oxygen supply in the brain is regulated from the site of increased oxygen exchange in the brain and its surrounding areas. The nature of the mechanisms that regulate the simultaneous oxygen supply at sites of increased oxygen exchange is also an important research topic at the level of whole brain regulation and at the level of more microscopic cell populations. As the weight of dumbbells lifted increases or the subject's muscle fatigue increases, the mechanisms that attempts to maintain COE homeostasis for the whole brain may be more likely to be strongly detected by vector method fNIRS. Thus, the closer to maximal voluntary muscle contraction, the more brain-muscle coordination may be required.

Compared with previous studies using verbal tasks (Kato et al., 2003; Yoshino and Kato, 2012), the present results suggest that, during muscle training, oxygen consumption and oxygen supply are controlled in areas of the cortex within a few centimeters square.

The FORCE, which is an oxygen-consuming response that occurs in a high neuroactivation site, has been observed in invasive human studies using optical imaging (Suh et al., 2006). However, it does not report that the phenomenon of oxygen supply surrounding the FORCE effect in the form of a doughnut has occurred, as obtained in this experiment. The strong and persistent FORCE effect on the homeostasis of oxygen exchange for the whole brain, as obtained in this experiment, may be a different brain mechanism from the temporary FORCE that has been previously reported (Kato, 2004, 2018).

It has been shown that γ-aminobutyric acid (GABA) concentrations during a motor learning task decrease in the left M1 and increase around it (Gudberg et al., 2012).

Studies using proton magnetic resonance spectroscopy have shown interactions between M1 and its surrounding sites in neurotransmitters (Umesawa et al., 2020; Maruyama et al., 2021). Although this method cannot measure intracellular and extracellular GABA separately, it could be used in conjunction with the vector method fNIRS to clarify the relationship between oxygen metabolism and GABA. However, because the dumbbell task is not a motor learning task, it does not necessarily elicit the same response as the strength training task. Further evidence and discussion of the physiological link between GABA and oxygen kinetics are needed.

### Advantages of vector-based analysis

The CORE model of 2-dimensional analysis of ΔO and ΔD was used to simultaneously generate functional distribution images using seven indices, including oxygen exchange.

The advantage of vector-based fNIRS is that multiple indices with different physiological significance can be compared to understand better neurovascular-coupling mechanisms and neuro-oxygen coupling that accompany neuroactivation. Conversely, these indices cannot be separately detected using the fMRI BOLD signal, which includes the effects of both oxyHb and deoxyHb changes (Yamamoto and Kato, 2002).

Unlike the other component images, the oxygen exchange images were able to indicate both high oxygen exchange sites, such as Phase 3 sites (common in the M1), and reduced oxygen exchange sites, such as Phase −1 (more common in the surrounding area), even though Δ*CBV* increased in the same way in both types of sites. The fact that the M1 site was most frequently a Phase 3 site (Δ*COE* increased more than Δ*CBV*) reflects the fact that the increase in Δ*D* attributable to oxygen consumption exceeds the increase in Δ*O* from oxygen supply. Together with *k*, the Δ*D* and Δ*COE* indices (reflecting oxygen consumption) increased most at the SMA for 0 kg, and at the M1 for 4.5 and 9.5 kg. However, the sites showing maximum increases in the Δ*O*, Δ*CBV*, and *L* indices (strongly affected by blood supply) did not coincide with the M1. Phase −1 is a non-activation phase, and I also observed that it did not reflect neuroactivation in a language task (Yoshino and Kato, 2012). In Phase −1, Δ*O* increases and Δ*D* decreases, which has been considered a typical fNIRS activation pattern; however, oxygen consumption is low in this phase, and it has become clear that it does not indicate a site where activation is strong. Based on our results, an increase in Δ*CBV* at a site does not necessarily imply an increase in oxygen demand. Variations were found in the oxygen exchange response concerning the amount of increase in Δ*O* or Δ*CBV*. For example, it was reported that, during a motor task, CMRO_2_ did not increase significantly at the M1, whereas CBF did (Vafaee et al., 2012); conversely, CBF did not increase significantly in the visual cortex, although CMRO_2_ did (Mintun et al., 2001).

### Possibilities and limitations of vector-based fNIRS

*Area*_*kL*_, which combines the indices *k* and *L*, proved to be useful for clearly imaging slight neuroactivation at the left M1 and SMA during a 0 kg task. Increased oxygen exchange, rather than the intensity of oxygen supply, coincided with the neuroactivation site. This result shows that the location and intensity of neuroactivation cannot be accurately estimated from the intensity of Hb concentration changes. Mintun et al. (2001) strongly suggested that increased CBF is caused by factors other than oxygen demand. The response at the M1 was in danger of being overlooked, masked by the intensity of Δ*O*. However, even small Hb changes, such as those from cognitive tasks, can provide dynamic oxygen consumption and oxygen supply images if *Area*_*kL*_ is used.

Because the dumbbell load in our study was sufficient to cause a systemic cardiovascular response, it is possible that systemic circulatory changes or scalp perfusion were included in the Δ*O* increases in the sites surrounding M1 (Kato et al., 1999). However, the donut-shaped excessive oxygen supply response generated in the area surrounding the neuroactivation site cannot be explained simply by changes in the systemic circulation. The most recent study (Kato, 2021) showed that vector-based fNIRS was less susceptible to artifacts from whole-body cardiovascular responses and motion. In other words, detecting localized increases in *k* may allow us to discount the broader influence of circulation and the autonomous nervous system.

I also observed an increase in *k* in a prior study of passive word listening (1.5-s tasks) that was unlikely to cause systemic circulation changes (Yoshino and Kato, 2012). It is important to note that the use of baseline normalization or motion correction to preprocess the data for analysis may distort the phase of oxygen exchange. For a rigorous study, it is interesting to use data from short channels where the distance between probes detects only the scalp. Even in such a comparative study, it may not be possible to conclude that systemic changes are not involved in brain responses.

When describing the exercise during validation, the loads used were 0, 4.5, and 9.5 kg, citing previous research showing that cortical potentials increase more with heavier loads (Tamaki et al., 1994). Due to the lack of information on the maximum voluntary muscle contraction of the participants' biceps curls, it is impossible to determine whether these loads were truly heavy for the participants. Bearability for loads varies from person to person; what may be light for one person may be heavy for another. It is necessary to study the COE distribution when using dumbbells of a weight that cannot be lifted and the relationship between maximal voluntary muscle contraction and oxygen exchange. By focusing on the strong FORCE effect and the surrounding oxygen supply response using the vector-based fNIRS, it may be possible to distinguish whether the subject's inability to lift the dumbbell is due to muscle fatigue or brain fatigue, or whether the patient is capable of lifting the dumbbell but does not intentionally try to do so.

## Conclusion

Vector-based fNIRS allows to image the spatial dissociation between oxygen consumption and supply in the brain during the dumbbell exercises. Based on seven indexes, vector-based fNIRS can image aspects of motor activation that other brain functional imaging modalities cannot detect. Moreover, vector-based fNIRS is a useful brain measurement method for understanding how increased oxygen exchange in M1 causes hypoxia and how the area surrounding the M1 provides fresh blood volume. The comparison of *k, L*, an index reflecting the local oxygen exchange distribution and its intensity obtained from vector-based fNIRS, with indices such as CBV, COE, OxyHb, DeoxyHb, CBF, and CMRO2 will be an important topic for future fNIRS studies to interpret oxygen dynamics better.

## Data availability statement

The raw data supporting the conclusions of this article will be made available by the author, without undue reservation.

## Ethics statement

The studies involving human participants were reviewed and approved by the Ethics Committee of KatoBrain Co. Ltd. The patients/participants provided their written informed consent to participate in this study.

## Author contributions

TK contributed to conception and design of the study, organized the data, and wrote the draft of the manuscript.

## Conflict of interest

Author TK was employed by KatoBrain Co., Ltd.

## Publisher's note

All claims expressed in this article are solely those of the authors and do not necessarily represent those of their affiliated organizations, or those of the publisher, the editors and the reviewers. Any product that may be evaluated in this article, or claim that may be made by its manufacturer, is not guaranteed or endorsed by the publisher.
